# ﻿Twenty-three new synonyms of the Eastern common groundhopper, *Tetrixjaponica* (Bolívar, 1887) (Orthoptera, Tetrigidae)

**DOI:** 10.3897/zookeys.1187.110067

**Published:** 2023-12-20

**Authors:** Ying Long, Caili Teng, Chaomei Huang, Rongjiao Zhang, Weian Deng, Liliang Lin

**Affiliations:** 1 Key Laboratory of Ecology of Rare and Endangered Species and Environmental Protection (Guangxi Normal University), Ministry of Education, Guilin, Guangxi 541006, China; 2 School of Chemistry and Bioengineering, Hechi University, Yizhou, Guangxi 546300, China; 3 Guangxi Key Laboratory of Rare and Endangered Animal Ecology, Guangxi Normal University, Guilin, Guangxi 541006, China; 4 College of Life Science, Guangxi Normal University, Guilin, Guangxi 541004, China; 5 College of Life Sciences, Shaanxi Normal University, Xi’an, Shaanxi 710062, China

**Keywords:** China, *Coptotettix*, *Euparatettix*, *Macromotettix*, taxonomy, Tetriginae

## Abstract

The Eastern common groundhopper, *Tetrixjaponica*, is a pygmy grasshopper species widely distributed in the Eastern Palearctic region, and shows a high degree of phenotypic variation. The classification of *Tetrixjaponica* is difficult and frequently involved errors. Among the many species of Tetrigidae that have been described in China within the last decades, many synonyms of *Tetrixjaponica* were found. The type specimens of many species deposited in the Chinese museums have been re-examined and as a result, *Tetrixjaponica* is systematically revised. Based on the results of this review, 23 new synonyms of *Tetrixjaponica* are proposed: *Coptotettixcircinihumerus* Zheng & Deng, 2004, **syn. nov.**; *Coptotettixemeiensis* Zheng, Lin & Zhang, 2012, **syn. nov.**; *Euparatettixrongshuiensis* Zheng, 2005, **syn. nov.**; *Euparatettixzayuensis* Zheng, Zeng & Ou, 2011, **syn. nov.**; *Macromotettixnigritubercle* Zheng & Jiang, 2006, **syn. nov.**; *Macromotettixyaoshanensis* Zheng & Jiang, 2000, **syn. nov.**; *Tetrixalbistriatus* Yao & Zheng, 2006, **syn. nov.**; *Tetrixalbomaculatus* Zheng & Jiang, 2006, **syn. nov.**; *Tetrixalbomarginis* Zheng & Nie, 2005, **syn. nov.**; *Tetrixcenwanglaoshana* Zheng, Jiang & Liu, 2005, **syn. nov.**; *Tetrixcliva* Zheng & Deng, 2004, **syn. nov**.; *Tetrixduolunensis* Zheng, 1996, **syn. nov.**; *Tetrixgrossovalva* Zheng, 1994, **syn. nov.**; *Tetrixjiuwanshanensis* Zheng, 2005, **syn. nov.**; *Tetrixlatipalpa* Cao & Zheng, 2011, **syn. nov.**; *Tetrixliuwanshanensis* Deng, Zheng & Wei, 2007, **syn. nov.**; *Tetrixqinlingensis* Zheng, Huo & Zhang, 2000, **syn. nov.**; *Tetrixrectimargina* Zheng & Jiang, 2004, **syn. nov.**; *Tetrixruyuanensis* Liang, 1998, **syn. nov.**; *Tetrixxianensis* Zheng, 1996, **syn. nov.**; *Tetrixxinchengensis* Deng, Zheng & Wei, 2007, **syn. nov.**; *Tetrixyunlongensis* Zheng & Mao, 2002, **syn. nov.**; *Tetrixzhoushanensis* Gao, Liu & Yin, 2022, **syn. nov.** It is expected that there will be the discoveries of more synonyms of this and other Tetriginae species from the Eastern Palearctic.

## ﻿Introduction

**Figure 1.**
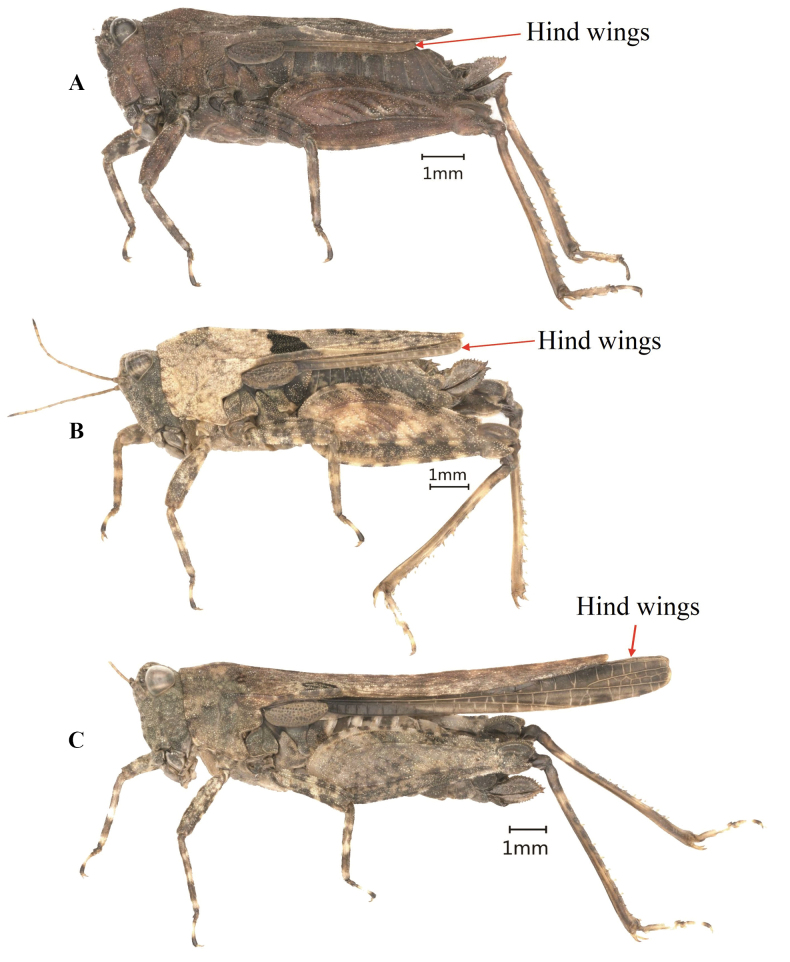
Dimorphism of *Tetrixjaponica* (Bolívar, 1887) **A, B** brachypronotal and brachypterous individuals **C** pauropronotal individual.

The Eastern groundhopper, *Tetrixjaponica* (Bolívar, 1887), is widely distributed in East Asia (China, Japan, North Korea, and Russia), and may be also present in Mongolia, Myanmar, Laos, and Vietnam. It is a very common species in China. *Tetrixjaponica* inhabits many different habitat types, from low grassland areas with moss to higher elevation areas such as hills and mountains. Its main foods are tender mosses and humus. *Tetrixjaponica* is a dimorphic species from the standpoint of wings and pronotum length (Deng 2021; Zhang et al. 2022). Within the same population, *T.japonica* can have brachypronotal and brachypterous individuals (Fig. 1A, B), macropronotal, and pauropronotal individuals (Fig. 1C). Brachypronotal and brachypterous individuals are those that have a short pronotum and hind wings. Their pronotum generally does not reach the apex of the hind femur and their hind wings do not reach or only slightly surpass the apex of the hind pronotal process. Macropronotal individuals are these with pronotum longer than the apex of the hind femur, but whose wings do not exceed the tip of the pronotum, while pauropronotal individuals are those with a prolonged pronotum and hind wings. The pronotum reaches the middle of the hind tibia and their hind wings extend beyond the pronotal apex (Devriese et al. 2023) and nearly reach the apex of the hind tibia.

Many new species of Tetrigidae have been described from China in the past (Liang and Zheng 1998; Zheng 2005a; Deng et al. 2007a; Deng 2016; Cigliano et al. 2022); however, many of them are known only from the type material and never recorded again. Tetriginae are an especially complicated subfamily from the standpoint of taxonomy, as its members lack most of clear traits present in other Tetrigidae subfamilies (Skejo and Gupta 2015; Tumbrinck 2019; Kasalo et al. 2023). Revisionary studies have recently discovered that species of some genera were described without clear traits (Adžić et al. 2020; Lu and Zha 2020; Wei and Deng 2023).

Because of the aforementioned, the aim of this study was to revise the type material deposited in the natural history museums of China and find which were *Tetrixjaponica* described under different names: we present 23 newly discovered synonyms of this species and analyze the probable causes of the description of so many synonyms. The goals include the determination of species variability and establishment of a good taxonomic practice (Lehmann et al. 2017) in Tetrigidae identification of species found in China.

## ﻿Materials and methods

### ﻿Taxonomy, nomenclature, terminology, and measurements

Taxonomy follows Orthoptera Species File [OSF] (Cigliano et al. 2022), a database of Orthoptera taxonomy. Nomenclature is in accordance with the International Code of the Zoological Nomenclature (ICZN 1999). Morphological terminology and landmark-based measurement method followed those used by Zheng (2005a), Deng et al. (2007a), Tumbrinck (2014, 2019), Muhammad et al. (2018), Tan and Artchawakom (2015), Devriese et al. (2023), and Zha et al. (2020). Measurements are given in millimeters (mm).

### ﻿Photography

Grasshopper specimens were examined using a Motic-SMZ-168 stereo-microscope and photographed using a KEYENCE VHX-600 Digital Microscope. All images were processed with Adobe photoshop CS 11.0.

### ﻿Type specimen depositories

The specimens examined in this study, including all holotypes and paratypes, have been deposited in the following institutions:

**BMSYU** Biology Museum of Sun Yat-sen University, Guangzhou, PR China;

**CLSGNU**College of Life Science, Guangxi Normal University, Guilin, China;

**EMHU** Entomological Museum of Hechi University, Hechi, China;

**IZSNU**Institute of Zoology, Shaanxi Normal University, Xi’an, China;

**MHNG**Muséum d’histoire naturelle, Geneve, Switzerland;

**MHU** Museum of Hebei University, Baoding, China.

## ﻿Results

### ﻿ Tetrix japonica

Taxon classificationAnimaliaOrthopteraTetrigidae

(Bolívar, 1887)

8F154C0E-F9A8-5B79-8917-16465FAAA700

Tettix japonicus : Bolívar, 1887: 263 [description] (holotype ♀, Japan, in MHNG).
Acrydium japonicum : Rehn 1902: 629; Kirby 1910: 45.
Tetrix japonica : Bey-Bienko 1934: 9; Bey-Bienko and Mistshenko 1951: 105; Yin 1984: 16; Blackith 1992: 183; Ichikawa 1993: 1; Paris 1993: 241; Storozhenko et al. 1994: 13; Ma and Zheng 1994: 445; Jiang and Zheng 1998: 340; Liang and Zheng 1998: 174; Kim and Kim 2004: 266; Jiang and Liang 2004: 204; Zheng 2005: 334a; Benediktov 2005; Deng et al. 2007a: 302; Tsurui Honma and Nishida 2010: 2; Cao and Zheng 2011: 738; Kim and Puskás 2012: 12; Xiao et al. 2012: 288; Zheng 2014: 56; Storozhenko et al. 2015: 167; Deng 2016: 250.

#### Previously reported synonyms.

*Tettixlongulus* Shiraki, 1906, *Tettixsibiricus* Bolívar, 1887, *Tetrixtrux* Steinmann, 1964.

#### Link.

https://orthoptera.speciesfile.org/otus/809028/overview.

#### Redescription.

**Figure 2.**
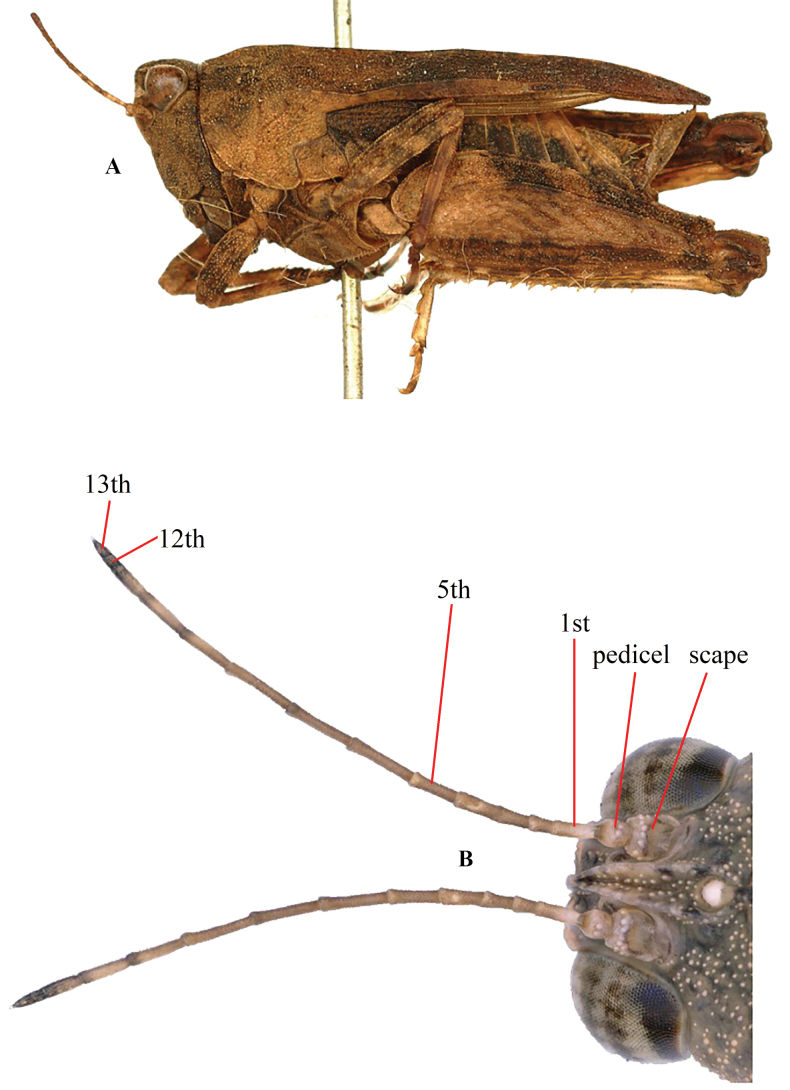
*Tetrixjaponica* (Bolívar, 1887) **A** syntype, lateral view (photograph Josef Tumbrinck) **B** antenna, 15-segmented (including scape, pedicel, 13-segmented flagellum).

**Female** (Figs 1, 2). Small size and short in brachypronotal and brachypterous individuals, or medium size and long in pauropronotal individuals. Body surface smooth and interspersed with granules.

***Head*.** Head and eyes not exserted above pronotal surface. In dorsal view, fastigium of vertex short; width of vertex between eyes generally wider than width of a compound eye (1.3–1.6 ×), sometimes 1.0 × (such as *T.albistriatus* syn. nov., *T.rectimargina* syn. nov.); anterior margin of fastigium truncated or slightly arcuate and slightly surpassing anterior margin of eye; median carina visible; lateral margins slightly turned backward; depressed on either side of median carina. In lateral view, frontal ridge and vertex forming an obtuse angle; frontal costa slightly straight above antennal groove, protruded anteriorly and broadly rounded between antennal grooves. In frontal view, frontal costa bifurcated above lateral ocelli, longitudinal furrow divergent between antennae, width of longitudinal furrow of frontal ridge narrower than antennal groove diameter. Antennae short, filiform, antennal grooves inserted between inferior margins of compound eyes, 15-segmented (including scape, pedicel, and a 13-segmented flagellum) (Fig. 2B), the 9^th^ and 10^th^ segment are the longest, ~ 4–5 × longer than its width. Eyes globose, lateral (paired) ocelli located in middle of compound eye height.

***Thorax.*** Pronotum slightly tectiform, its surface smooth and interspersed with dense granules. In dorsal view, anterior margin of pronotum generally truncate, sometimes slightly angular protruding; lateral carinae of prozona generally parallel or sometimes slightly constricted backwards; median carina low and full length entire; humeral angle obtuse; hind pronotal process narrow and long, pronotal apex either generally slightly not reaching or sometimes reaching apex of hind femur in brachypronotal and brachypterous individuals (Fig. 2C) or distinctly surpassing apex of hind femur and reaching approximately the middle of hind tibia in pauropronotal individuals (Fig. 2A, B). In profile, median carina of pronotum slightly straight or slightly arch-like before the shoulders and straight behind the shoulders. Posterior margins of lateral lobes of pronotum with ventral sinus and tegminal sinus. Posterior angles of lateral lobes turned downwards, generally narrow and rounded or sometimes subtruncately at apex. Tegmina long, ovate, apex rounded. Hind wings either slightly not reaching or reaching or slightly surpassing apex of hind pronotal process in brachypronotal and brachypterous individuals (Fig. 2C) or distinctly surpassing apex of hind pronotal process and nearly reaching of apex of hind tibia in pauropronotal individuals (Fig. 2A, B).

***Legs.*** Fore and middle femora slightly compressed and margins finely serrated, ventral margins of middle femora straight or slightly undulated, middle femur slightly narrower than or equal to or slightly wider than visible part of tegmen in width. Hind femora robust and short, 2.8–3.4 × as long as wide; margins finely serrated; antegenicular denticles acute and genicular denticles obtuse. Outer side of hind tibia with seven or eight spines, inner side with six or seven spines. Length of first segment of posterior tarsi longer than third, three pulvilli of first segment of posterior tarsi increased in turn, apices of all pulvilli acute.

***Abdomen.*** Ovipositor narrow and long, length of upper valvulae 3.0 × its width, upper and lower valvulae with slender saw-like teeth. Length of subgenital plate longer than its width, middle of posterior margin of subgenital plate triangularly projecting.

***Coloration.*** Body yellow brown or brown or dark brown; antennae brown; dorsum of pronotum with two black spots behind the shoulders or with two black spots before the shoulders and behind the shoulders respectively or without black spot. Hind femora brown, outer side with two inconspicuous blackish spots in some individuals. Hind tibia yellow brown or brown or dark brown.

**Male.** Similar to female, but smaller and narrower. Width of vertex between eyes generally 1.2–1.5 × or sometimes equal to width of compound eye; dorsal margins straight and ventral margins of middle femora straight or slightly undulated, middle femur generally wider than or sometimes equal to visible part of tegmen in width. Subgenital plate short, cone-shaped, apex bifurcated.

#### Measurements (mm).

See Table 1. Length of body: ♂ 6.0–10.0 (brachypronotal individuals) or 8.0–10.0 (pauropronotal individuals), ♀ 9.0–12.0 (brachypronotal individuals) or 8.5–11.0 (pauropronotal individuals); length of pronotum: ♂ 6.0–8.5 (brachypronotal individuals) or 6.0–11.0 (pauropronotal individuals), ♀ 7.5–9.0 (brachypronotal individuals) or 7.0–13.0 (pauropronotal individuals); length of hind femur: ♂ 5.0–6.0 (brachypronotal individuals) or 5.0–5.5 (pauropronotal individuals), ♀ 5.0–8.1(brachypronotal individuals) or 6.0–7.0 (pauropronotal individuals).

**Table 1.** Measurements for the type specimens of synonyms of *T.japonica*.

Species	V/E		LB (in mm)		LP (in mm)		LF (in mm)		M/T		LH/WH
	female	male	female	male	female	male	female	male	female	male	
C.circinihumerus**	1.6	–	10.3	–	9.2	–	6.0	–	< 1	–	–
C.emeiensis*	1.3	–	10.2	–	8.0	–	8.1	–	< 1	–	–
E.rongshuiensis*	–	1.1	–	8.0	–	6.0	–	5.0	–	> 1	–
E.zayuensis*	1.3	–	10.2	–	8.4	–	6.0	–	> 1	–	–
M.nigritubercle*	1.3	1.3	1.0	8.0	9.0	7.0	5.0	5.0	= 1	> 1	–
M.yaoshanensis*	–	1.0	11.0	8.0	9.0	7.0	6.0	5.5	= 1	> 1	–
T.albistriatus*	1.0	1.0	12.0	10.0	8.5	8.0	7.0	5.0	= 1	= 1	–
T.albomaculatus*	–	1.0	–	9.0	–	8.5	–	5.5	–	= 1	–
T.albomarginis*	–	1.4	–	6.0	–	6.0	–	5.0	–	= 1	–
T.cenwanglaoshana**	1.6	–	9.0	–	10.5	–	6.0	–	< 1	–	–
T.cliva*	–	1.0	–	7.2	–	7.5	–	5.5	–	= 1	3.0
T.duolunensis*	1.6	1.2	9.0	7.0	8.0	6.0	6.0	5.0	= 1	> 1	2.6
T.grossovalva**	1.5	–	11.0	–	13.0	–	7.0	–	–	–	3.5
T.jiuwanshanensis**	1.6	1.5	11.0	8.0	12.0	10.0	6.0	5.0	= 1	> 1	–
T.latipalpa*	1.7	–	10.0	–	7.5	–	7.0	–	= 1	–	–
T.liuwanshanensis*	–	1.1	–	9.5	–	8.0	–	5.5	–	> 1	2.8
T.qinlingensis**	1.6	1.5	11.0	10.0	13.0	11.0	6.5	5.5	< 1	= 1	3.0
T.rectimargina*	1.0	–	11.0	–	8.0	–	7.0	–	> 1	–	3.3
T.ruyuanensis*	1.5	1.5	11.0	9.0	9.0	7.0	7.0	5.0	> 1	> 1	2.8
T.xianensis**	1.1	–	8.5	8.0	7.0	6.0	6.0	5.0	< 1	–	2.5
T.xinchengensis**	–	1.0	11.0	–	9.0	–	7.0	–	–	> 1	3.0
T.yunlongensis*	–	1.4	–	7.5	–	6.0	–	6.0	–	= 1	2.6
T.zhoushanensis*	1.1	1.0	9.9	7.8	8.5	6.3	6.2	6.0	= 1	= 1	3.0

Note: V/E: Vertex wide/eye diameter; LB: Length of body; LP: Length of pronotum; LF: Length of hind femur; M/T: Width of midfemur/width of visible part of tegmina; LH/WH: Length of hind femur/width of hind femur. –: not described or illustrated in the original descriptions of species. *: brachypronotal; **: pauropronotal.

#### Diagnosis.

*Tetrixjaponica* can be differentiated from all the other Tetrigidae of China, North Korea, and Japan by the following set of the traits: the head not exserted above the upper level of the pronotum (strongly exserted in *Euparatettix*, *Ergatettix*, and *Bannatettix*); in lateral view, frontal ridge and vertex forming an obtuse angle (in lateral view, frontal ridge and vertex forming rounded shape in *Coptotettix* and *Hedotettix*); fastigium of vertex in lateral view angulate, not much produced in front of eyes (fastigium of vertex in lateral view oblique, considerably produced in front of eyes in *Clinotettix*); anterior margin of the vertex truncated, weakly arcuate (strongly angular in *Tetrixbipunctata*, *Tetrixsubulate*, *Tetrixsimulans*); anterior margin of pronotum truncate, weakly angular protruding (strongly angular in *Tetrixtartara*); tegmenula and alae present (absent in *Formosatettix*, *Aalatettix*); alae > 2 × longer than tegmenula (short in *Tetrixbipunctata*, *Alulatettix*).

*Tetrixjaponica* is most similar to *Tetrixtenuicornis* (Sahlberg, 1893) from the Western Palearctic from which it differs in its pronotum slightly tectiform, median carina of pronotum low, not lamellar (vs pronotum distinctly tectiform, median carina of pronotum high, lamellar in *T.tenuicornis*).

#### A catalog of synonyms.

(map of all the type localities of all the synonyms in Fig. 16).

**Figure 3.**
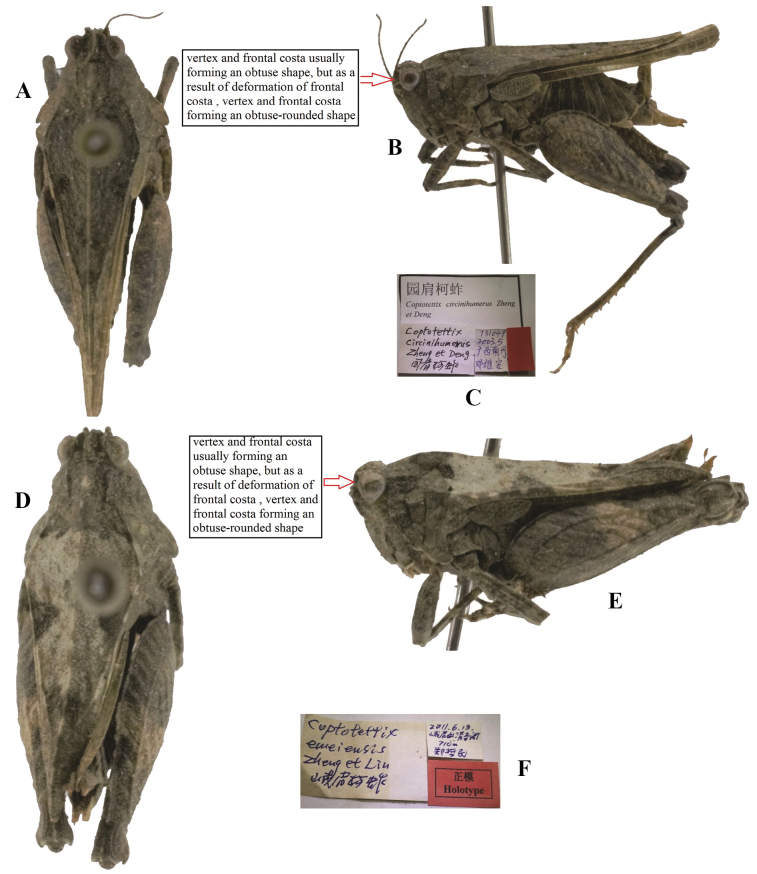
*Tetrixjaponica* (Bolívar, 1887) **A–C** holotype of *Coptotettixcircinihumerus* Zheng & Deng, 2004, syn. nov. **A** dorsal view **B** lateral view **C** labels **D–F** holotype of *Coptotettixemeiensis* Zheng, Lin & Zhang, 2012, syn. nov. **D** dorsal view **E** lateral view **F** labels.

*Coptotettixcircinihumerus* Zheng & Deng, 2004a: 79 [description] (holotype ♀, China: Guangxi prov., Nanda County, in IZSNU, examined); Zheng 2005a: 237; Deng et al. 2007a: 214; Zheng et al. 2013: 22; Deng 2016: 197. syn. nov. (Fig. 3A–C).

*Coptotettixemeiensis* Zheng, Lin & Zhang, 2012: 2554 [description] (holotype ♀, China: Sichuan prov., Emeishan City, in IZSNU, examined); Zheng et al. 2013: 22; Deng 2016: 197. syn. nov. (Fig. 3D–F).

**Figure 4.**
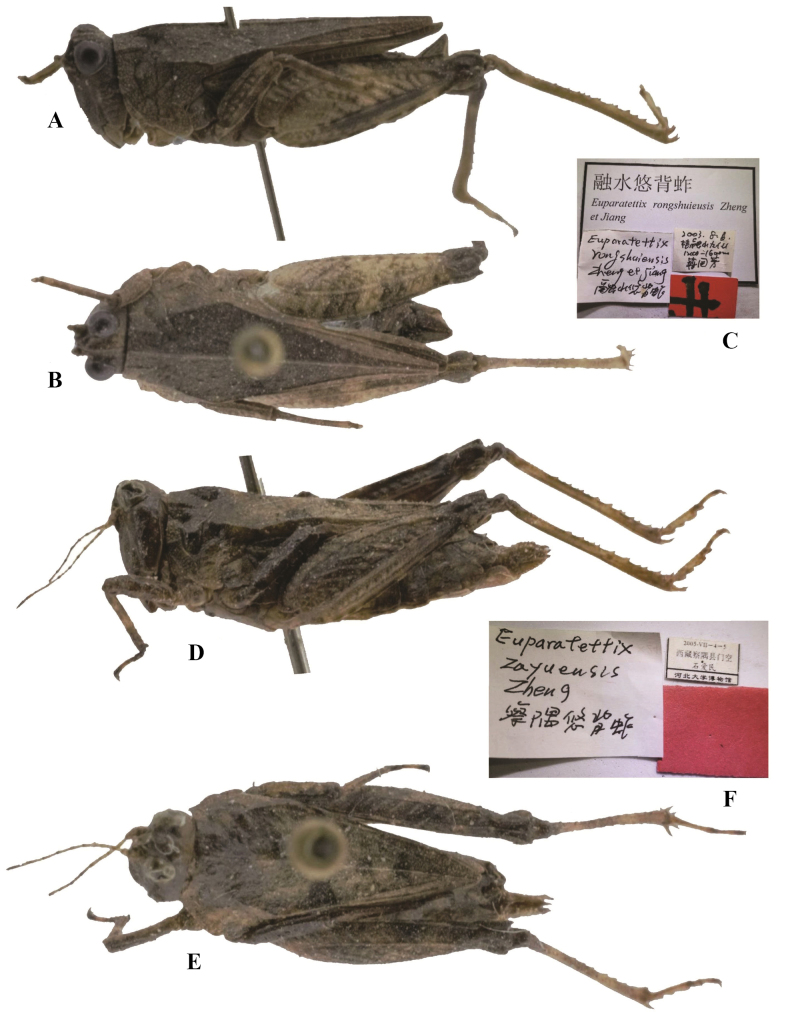
*Tetrixjaponica* (Bolívar, 1887) **A–C** holotype of *Euparatettixrongshuiensis* Zheng, 2005, syn. nov. **A** lateral view (The pin passes through the right side of the thorax from the shoulder of pronotum, which tends to push the pronotum down. This elevates the previously non-protruding head) **B** dorsal view **C** labels **D–F** holotype of *Euparatettixzayuensis* Zheng, Zeng & Ou, 2011, syn. nov. **D** lateral view (The pin passes through the right side of the thorax from the shoulder of pronotum, which tends to push the pronotum down. This elevates the previously unprotruding head) **E** dorsal view **F** labels.

*Euparatettixrongshuiensis* Zheng, 2005a: 387 [description] (holotype ♂, China: Guangxi prov., Rongshui County, in IZSNU, examined); Zheng 2005b: 99; Deng et al. 2007a: 444; Deng 2016: 294. syn. nov. (Fig. 4A–C)

*Euparatettixzayuensis* Zheng, Zeng & Ou, 2011: 387 [description] (holotype ♀, China: Xizang autonomous region, Zayu County, Menkong, in IZSNU, examined); Deng 2016: 293. syn. nov. (Fig. 4D–F).

**Figure 5.**
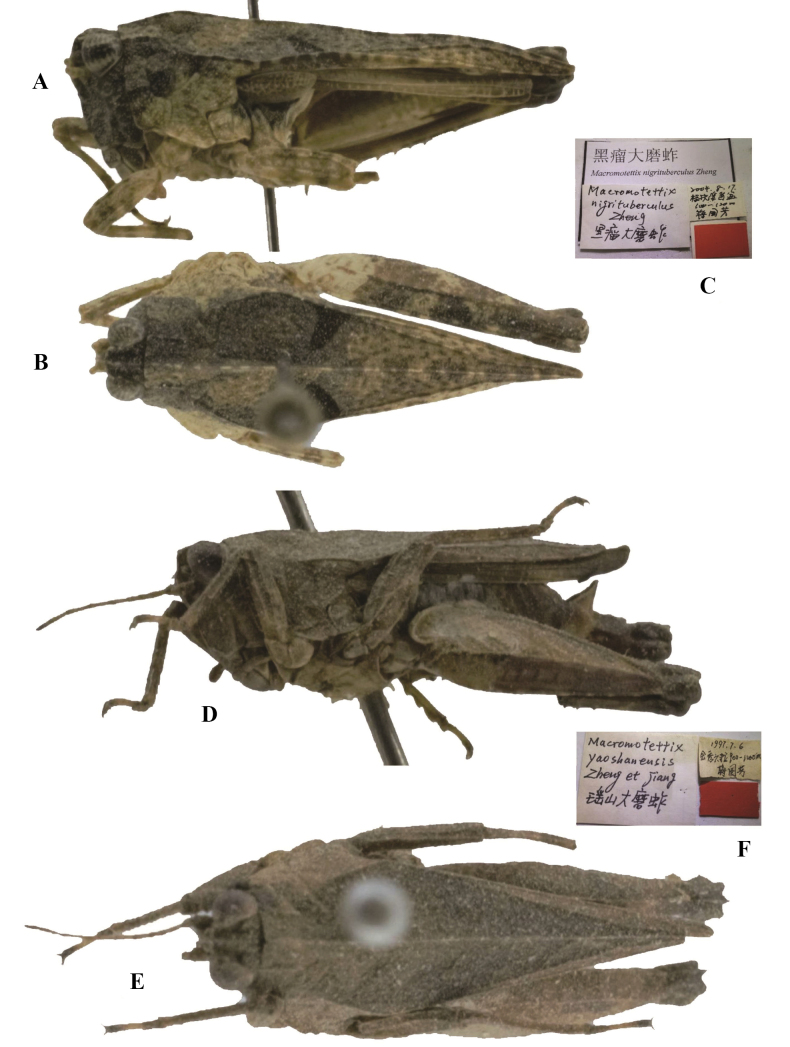
*Tetrixjaponica* (Bolívar, 1887) **A–C** holotype of *Macromotettixnigritubercle* Zheng & Jiang, 2006, syn. nov. **A** lateral view **B** dorsal view **C** labels **D–F** holotype of *Macromotettixyaoshanensis* Zheng & Jiang, 2000, syn. nov. **D** lateral view **E** dorsal view **F** labels.

*Macromotettixnigritubercle* Zheng & Jiang, 2006: 141[description] (holotype ♀, China: Guangxi prov., Fusui County, Bapan, in IZSNU, examined); Deng et al. 2007a: 136; Deng 2016: 135; Deng, Xin & Chen, 2018: 423. syn. nov. (Fig. 5A–C).

*Macromotettixyaoshanensis* Zheng & Jiang, 2000: 403 [description] (holotype ♂, China: Guangxi prov., Jinxiu County, Liula, in IZSNU, examined); Zheng 2005a: 143; Deng et al. 2007a: 132; Deng 2016: 130; Deng et al. 2018: 423. syn. nov. (Fig. 5D–F).

**Figure 6.**
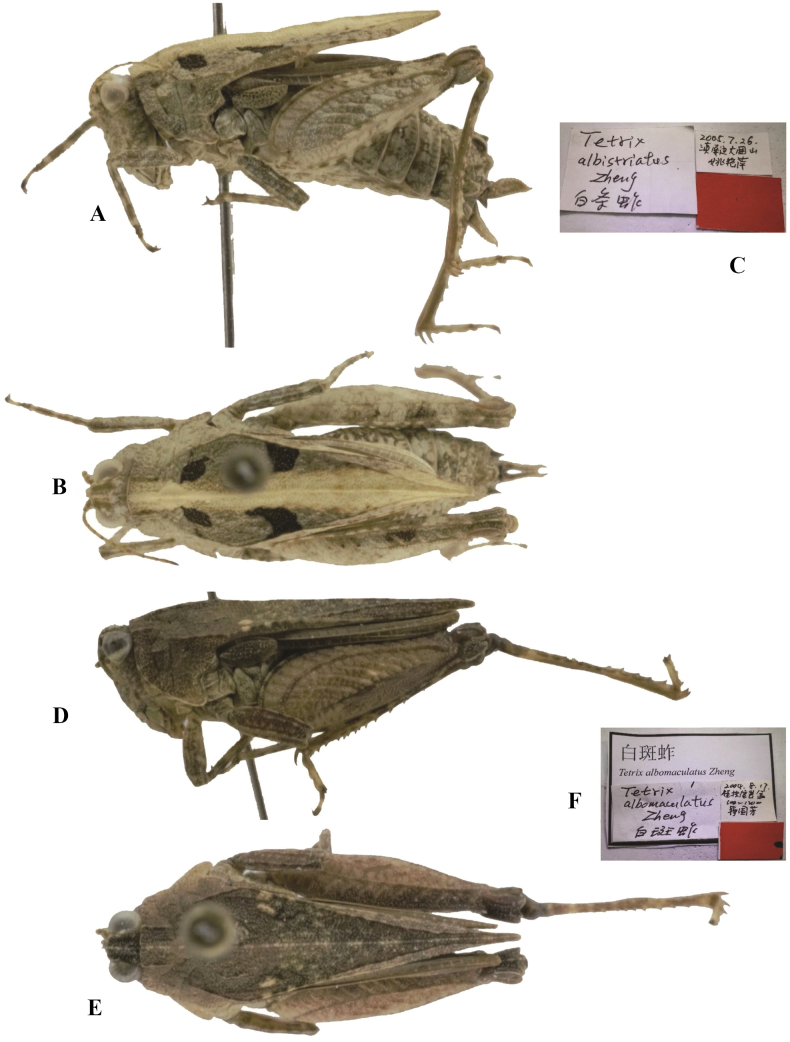
*Tetrixjaponica* (Bolívar, 1887) **A–C** holotype of *Tetrixalbistriatus* Yao & Zheng, 2006, syn. nov. **A** lateral view **B** dorsal view **C** labels **D–F** holotype of *Tetrixalbomaculatus* Zheng & Jiang, 2006, syn. nov. **D** lateral view **E** dorsal view **F** labels.

*Tetrixalbistriatus* Yao & Zheng, 2006: 824 [description] (holotype ♂, China: Yunnan prov., Pingbian County, Daweishan, in IZSNU, examined); Deng 2016: 245. syn. nov. (Fig. 6A–C).

*Tetrixalbomaculatus* Zheng & Jiang, 2006: 142 [description] (holotype ♂, China: Guangxi prov., Fusui County, Bapan, in IZSNU, examined); Deng et al. 2007a: 286; Deng 2016: 242. syn. nov. (Fig. 6D–F).

**Figure 7.**
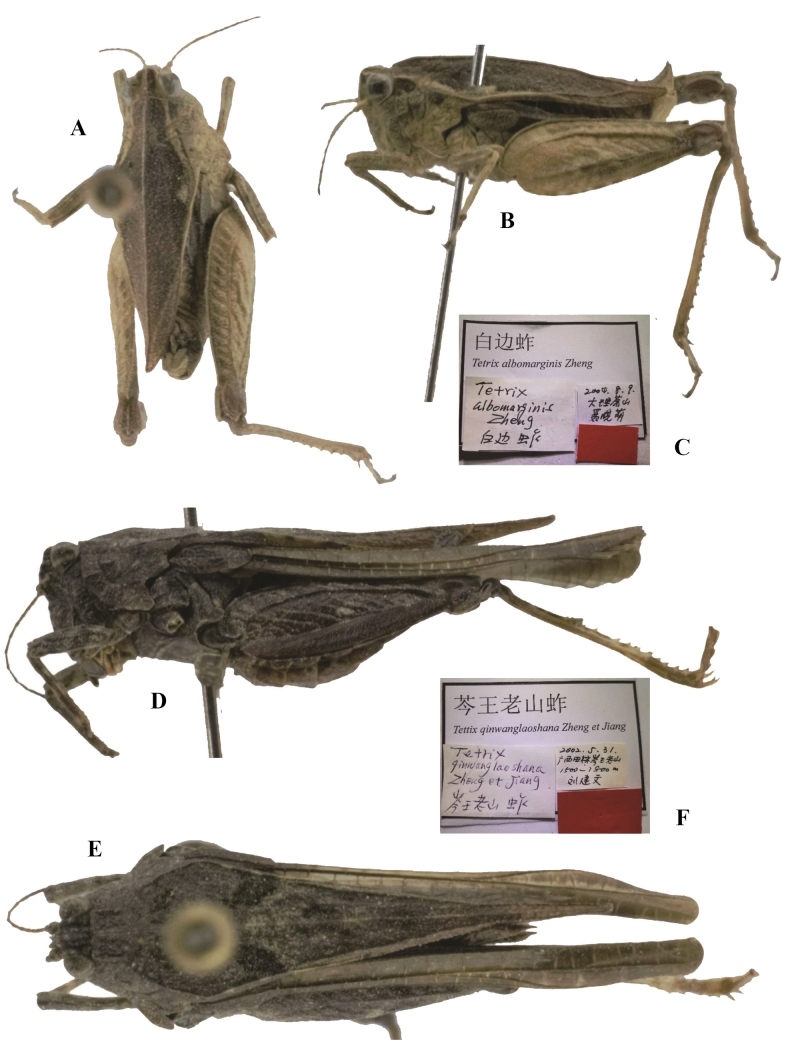
*Tetrixjaponica* (Bolívar, 1887) **A–C** holotype of *Tetrixalbomarginis* Zheng & Nie, 2005, syn. nov. **A** dorsal view **B** lateral view **C** labels **D–F** holotype of *Tetrixcenwanglaoshana* Zheng, Jiang & Liu, 2005, syn. nov. **D** lateral view **E** dorsal view **F** labels.

*Tetrixalbomarginis* Zheng & Nie, 2005: 582 [description] (holotype ♂, China: Yunnan prov., Dali County, Cangshan, in IZSNU, examined); Deng et al. 2007a: 300; Deng 2016: 245. syn. nov. (Fig. 7A–C).

*Tetrixcenwanglaoshana* Zheng, Jiang & Liu, 2005: 181 [description] (holotype ♀, China: Guangxi prov., Tianlin County, Cenwanglaoshan, in IZSNU, examined). syn. nov. (Fig. 7D–F).

**Figure 8.**
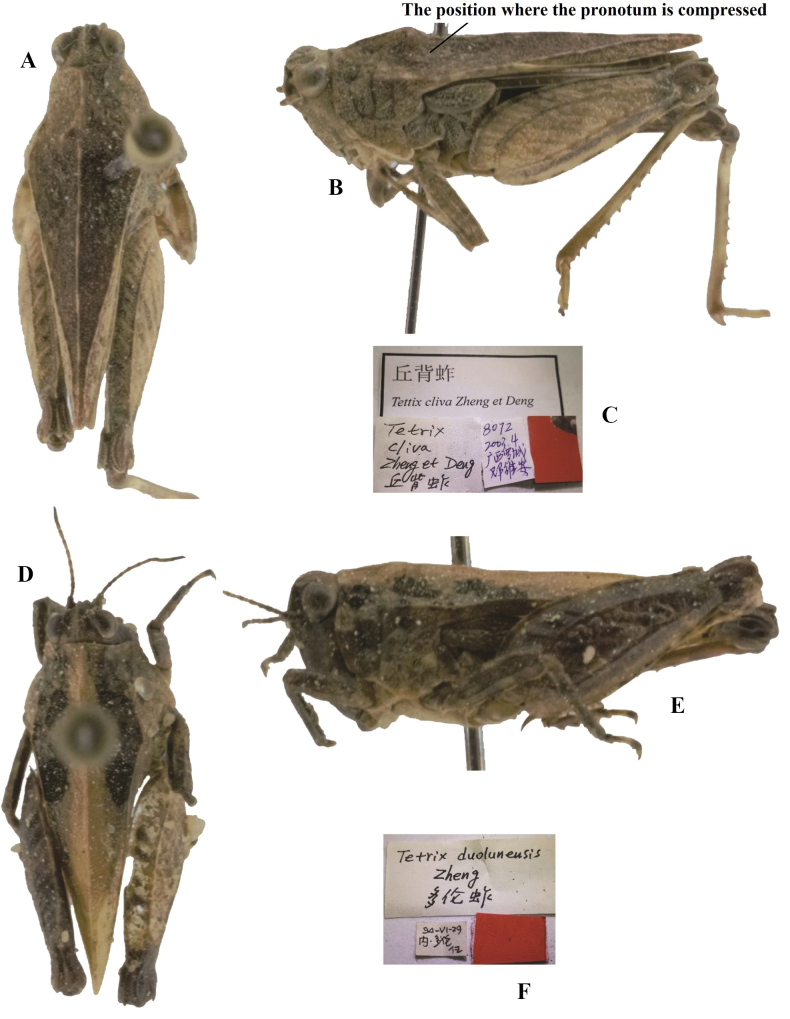
*Tetrixjaponica* (Bolívar, 1887) **A–C** holotype of *Tetrixcliva* Zheng & Deng, 2004, syn. nov. **A** dorsal view **B** lateral view **C** labels **D–F** holotype of *Tetrixduolunensis* Zheng, 1996, syn. nov. **D** dorsal view **E** lateral view **F** labels.

*Tetrixcliva* Zheng & Deng, 2004b: 97 [description] (holotype ♂, China: Guangxi prov., Luocheng County, in IZSNU, examined); Zheng 2005a: 322; Deng et al. 2007a: 291a; Deng 2016: 251 (*Tetrixcliva* Zheng & Deng, 2004 = *Tetrixruyuanensis* Liang, 1998, proposed in unpublished PhD Dissertation). syn. nov. (Fig. 8A–C).

*Tetrixduolunensis* Zheng, 1996: 178 [description] (holotype ♀, China: Inner Mongolia autonomous region, Duolun County, in IZSNU, examined); Zheng 2005c: 106; Deng 2016: 244. syn. nov. (Fig. 8D–F).

**Figure 9.**
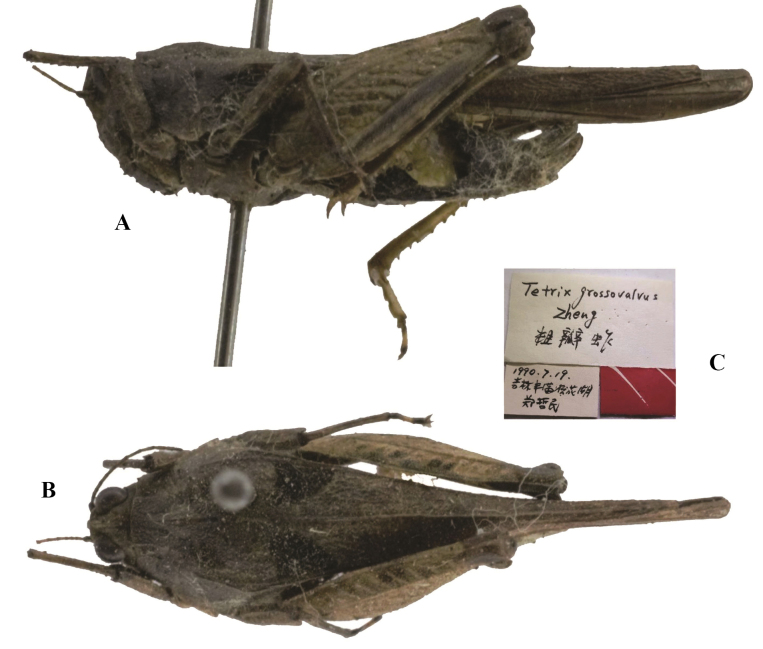
*Tetrixjaponica* (Bolívar, 1887). Holotype of *Tetrixgrossovalva* Zheng, 1994, syn. nov. **A** lateral view **B** dorsal view **C** labels.

*Tetrixgrossovalva* Zheng, 1994: 147 [description] (holotype ♀, China: Jilin prov., Fongman County, Songhuahu, in IZSNU, examined); Liang and Zheng 1998: 148; Zheng 2005c: 99; Deng 2016: 218. syn. nov. (Fig. 9).

**Figure 10.**
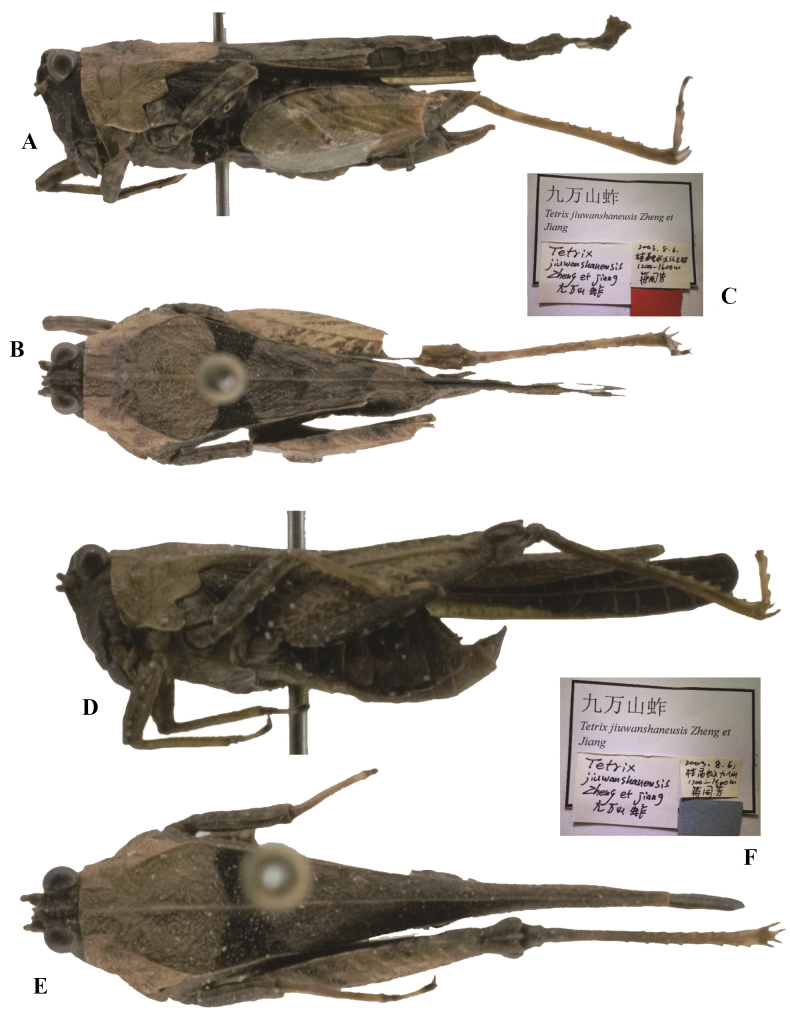
*Tetrixjaponica* (Bolívar, 1887). *Tetrixjiuwanshanensis* Zheng, 2005, syn. nov. **A** holotype, lateral view **B** holotype, dorsal view **C, F** labels **D** allotype, lateral view **E** allotype, dorsal view.

*Tetrixjiuwanshanensis* Zheng, 2005a: 274 [description] (holotype ♀, China: Guangxi prov., Rongshui County, in IZSNU, examined); Zheng 2005c: 100; Deng et al. 2007a: 250; Deng 2016: 220. syn. nov. (Fig. 10).

**Figure 11.**
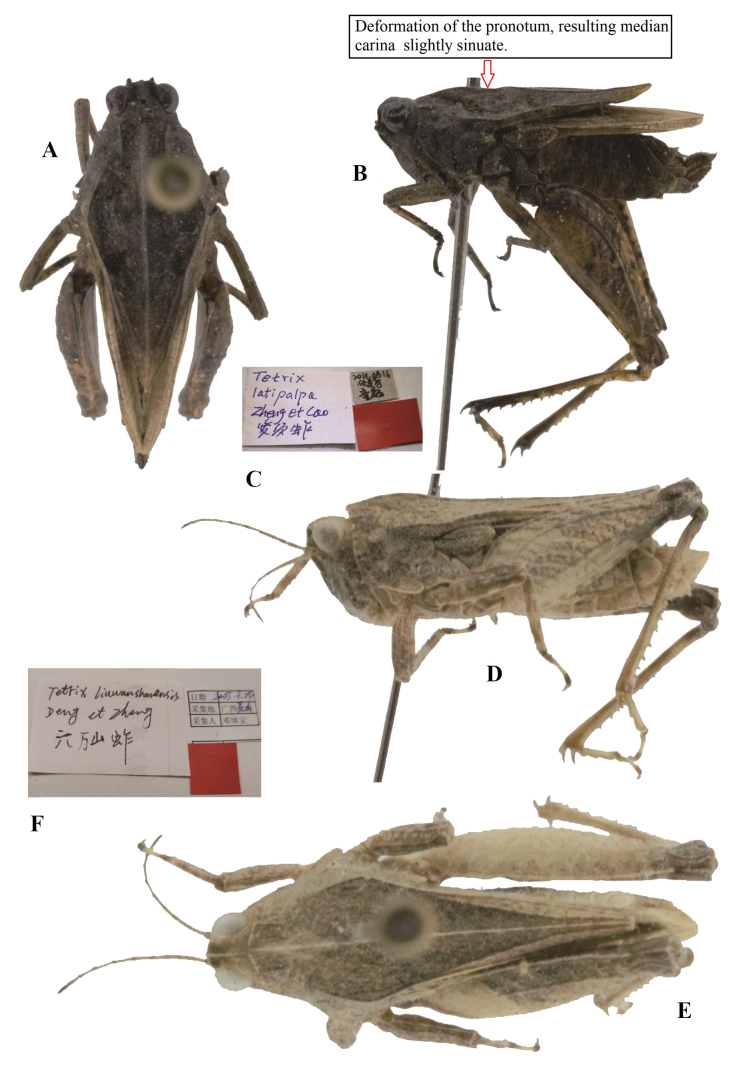
*Tetrixjaponica* (Bolívar, 1887) **A–C** holotype of *Tetrixlatipalpa* Cao & Zheng, 2011, syn. nov. **A** dorsal view **B** lateral view **C** labels **D–F** holotype of *Tetrixliuwanshanensis* Deng, Zheng & Wei, 2007, syn. nov. **D** lateral view **E** dorsal view **F** labels.

*Tetrixlatipalpa* Cao & Zheng, 2011: 739 [description] (holotype ♂, China: Sichuan prov., Emeishan County, Mt. Emei, in IZSNU, examined); Deng 2016: 249. syn. nov. (Fig. 11A–C).

*Tetrixliuwanshanensis* Deng, Zheng & Wei, 2007b: 294 [description] (holotype ♂, China: Guangxi prov., lingshan County, Liuwanda Mountain, in IZSNU, examined); Deng et al. 2007a: 277; Deng 2016: 238. syn. nov. (Fig. 11D–F).

**Figure 12.**
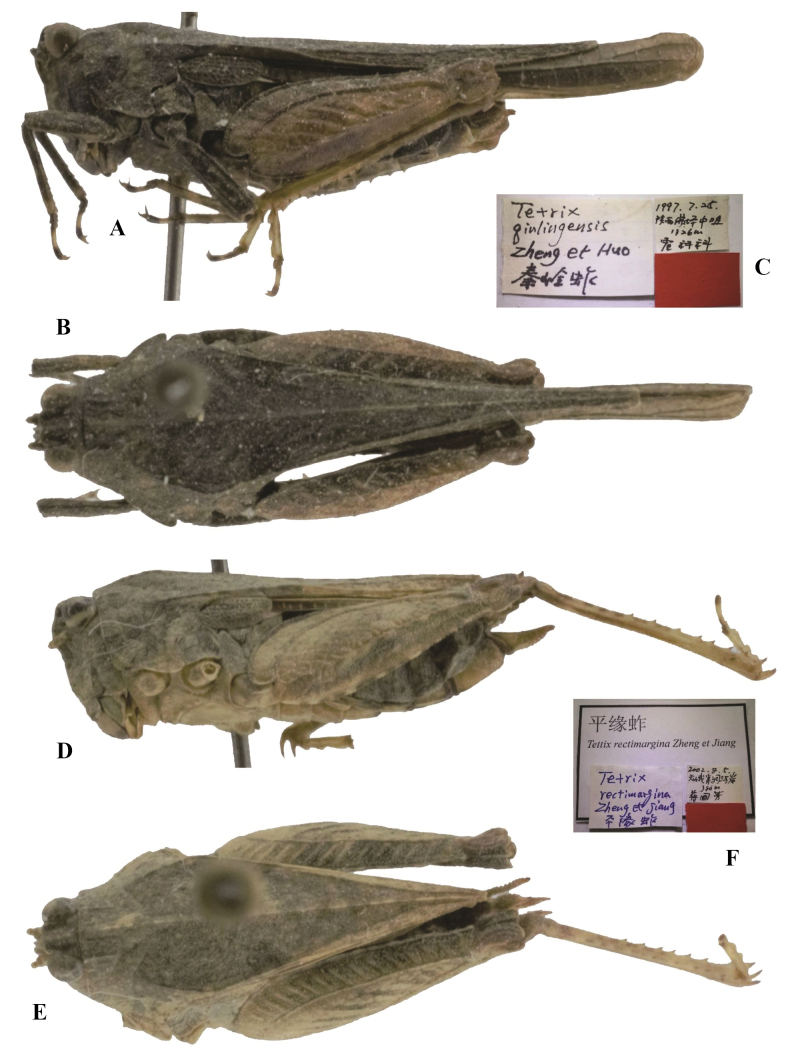
*Tetrixjaponica* (Bolívar, 1887) **A–C** holotype of *Tetrixqinlingensis* Zheng, Huo & Zhang, 2000, syn. nov. **A** lateral view **B** dorsal view **C** labels **D–F** holotype of *Tetrixrectimargina* Zheng & Jiang, 2004, syn. nov. **D** lateral view **E** dorsal view **F** labels.

*Tetrixqinlingensis* Zheng, Huo & Zhang, 2000: 238 [description] (holotype ♀, China: Shaanxi prov., Foping County, Zhongzui, in IZSNU, examined); Zheng 2005a: 275; Deng et al. 2007a: 251; Deng 2016: 220. syn. nov. (Fig. 12A, B).

*Tetrixrectimargina* Zheng & Jiang, 2004: 3 [description] (holotype ♀, China: Guangxi prov., Tian’e County, Buliu River, in IZSNU, examined); Zheng 2005a: 308; Deng et al. 2007a: 273; Deng 2016: 234. syn. nov. (Fig. 12D–F).

**Figure 13.**
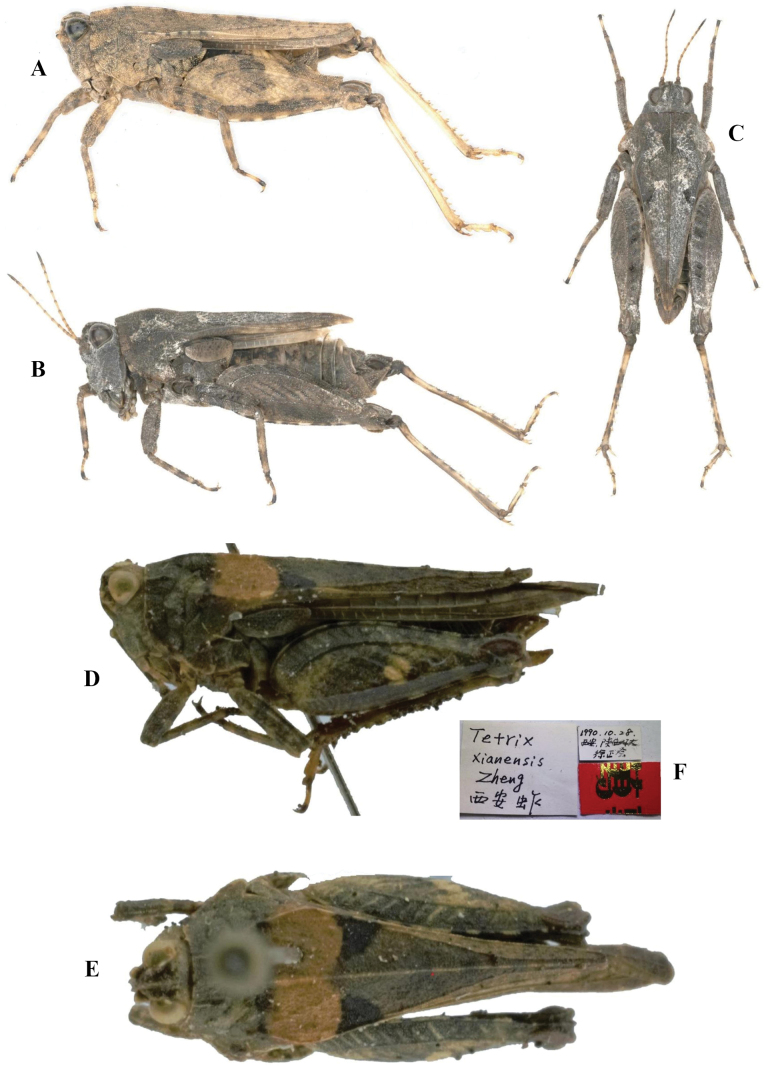
*Tetrixjaponica* (Bolívar, 1887) **A–C** topotype of *Tetrixruyuanensis* Liang, 1998, syn. nov. **A** lateral view, pronotum with nearly straight median carina **B** lateral view, pronotum with arcuate median carina before shoulders **C** dorsal view **D–F** holotype of *Tetrixxianensis* Zheng, 1996, syn. nov. **D** lateral view **E** dorsal view **F** labels.

*Tetrixruyuanensis* Liang, 1998: 174 [description] (holotype ♀, China: Guangdong prov., Ruyuan County, Tianjingshan, in BMSYU, not examined); Zheng 2005a: 307; Deng et al. 2007a: 303; Deng 2016: 251. syn. nov. (Fig. 13A, B).

*Tetrixxianensis* Zheng, 1996: 177 [description] (holotype ♀, China: Shaanxi prov., Xi’an City, Shaanxi Normal University, in IZSNU, examined); Zheng 2005a: 283; Deng 2016: 226. syn. nov. (Fig. 13D–F).

**Figure 14.**
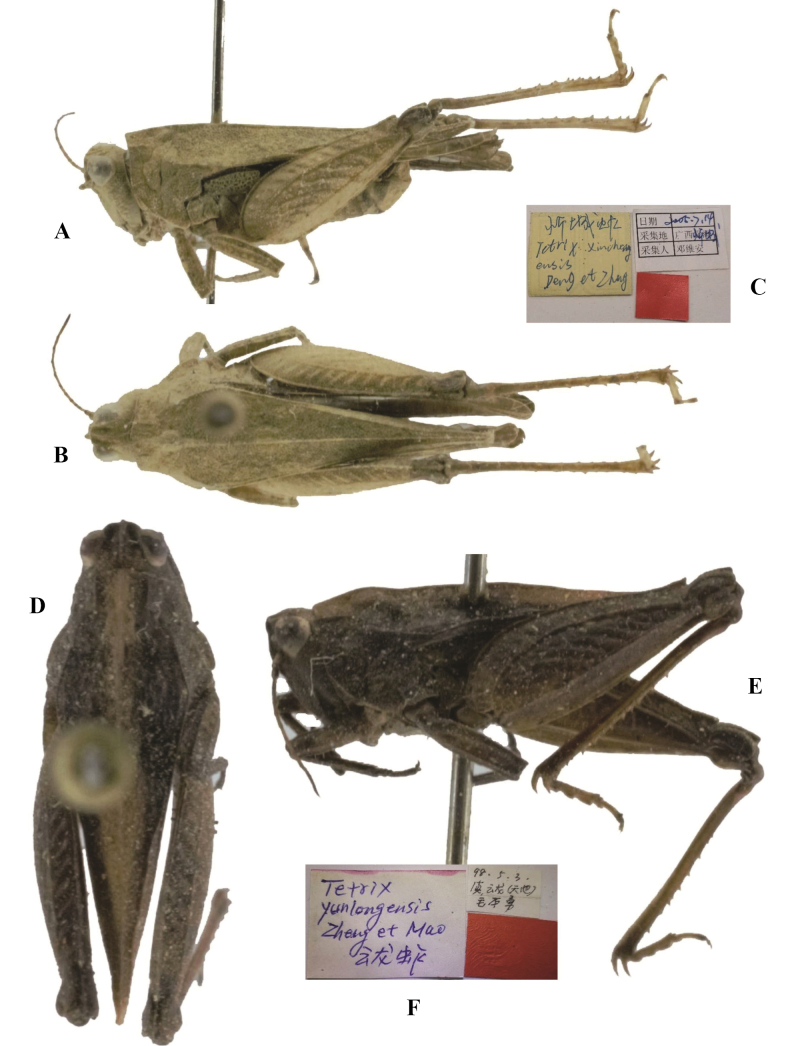
*Tetrixjaponica* (Bolívar, 1887) **A–C** topotype of *Tetrixxinchengensis* Deng, Zheng & Wei, 2007, syn. nov. **A** lateral view **B** dorsal view **C** labels **D–F** holotype of *Tetrixyunlongensis* Zheng & Mao, 2002, syn. nov. **D** lateral view **E** dorsal view **F** labels.

*Tetrixxinchengensis* Deng, Zheng & Wei, 2007a: 289[description] (holotype ♂, China: Guangxi prov., Xincheng County, in IZSNU, examined); Deng 2016: 242. syn. nov. (Fig. 14A, B).

*Tetrixyunlongensis* Zheng & Mao, 2002: 91[description] (holotype ♂, China: Yunnan prov., Yunlong County, in IZSNU, examined); Zheng 2005a: 311; Deng et al. 2007a: 275; Deng 2016: 236. syn. nov. (Fig. 14D, F).

**Figure 15.**
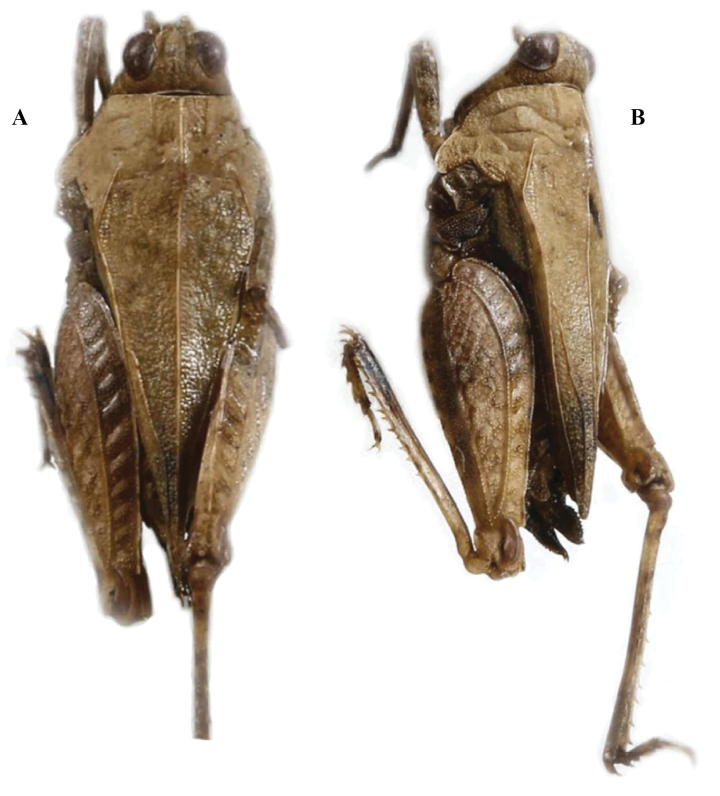
*Tetrixjaponica* (Bolívar, 1887). Holotype of *Tetrixzhoushanensis*, Gao, Liu & Yin, 2022, syn. nov. **A** dorsal view **B** lateral view (photo Gao et al.).

*Tetrixzhoushanensis* Gao, Liu & Yin, 2022: 347 [description] (holotype ♂, China: Zhejiang prov., Zhoushan City, in MHU, not examined). syn. nov. (Fig. 15).

#### Type material examined.

Type material of *Tetrixjaponica* was examined from the photographs of the syntype (♀, brachypronotal and brachypterous specimen, in MHNG, photographs by Josef Tumbrinck), available online in OSF (Fig. 2A).

#### Other material of *Tetrixjaponica* examined.

11♂23♀, China: Sichuan prov., Emeishan City, 29 July 2018, in EMHU; 17♂13♀, China: Xizang, Zayu County, 25 June 2019, in EMHU; 33♂19♀, China: Guangxi prov., Fusui County, Bapan, 17 August 2014, in EMHU; 24♂23♀, China: Guangxi prov., Jinxiu County, 26 July 2021, in EMHU; 37♂20♀, China: Yunnan prov., Pingbian County, Daweishan, 26 July 2020, in EMHU; 55♂27♀, China: Guangxi prov., Tianlin County, Cenwanglaoshan, 25 May 2018, in EMHU; 52♂38♀, China: Guangxi prov., Longzhou County, nonggang, 18 July 2023, in CLSGNU; 47♂58♀, China: Guangxi prov., Luocheng County, Jiuwanshan, 21 August 2022, in EMHU; 7♂8♀, China: Inner Mongolia autonomous region, Duolun County, 09 August 2019, in EMHU; 27♂32♀, China: Guangxi prov., Rongshui County, 06 August 2003, in EMHU; 17♂21♀, China: Guangxi prov., lingshan County, Liuwanda Mountain, 24 August 2022, in CLSGNU; 20♂8♀, China: Shaanxi prov., Foping County, 28 July 2022, in CLSGNU; 11♂15♀, China: Guangdong prov., Ruyuan County, Tianjingshan, 20 August 2022, in CLSGNU; 17♀10♂, China: Liaoning prov., Benxi County, Tanggou, 27 July 2023 in CLSGNU; 22♀22♂, China: Jilin prov., Changbai County, Changbai, 31 July 2023 in CLSGNU; 14♀23♂, China: Heilongjiang prov., Mudanjiang City, Mudanfen, 3 August 2023 in CLSGNU; 29♀13♂, China: Heilongjiang prov., Yichun City, Jiayin County, 6 August 2023 in CLSGNU; 22♀11♂, China: Heilongjiang prov., Xinlin County, Xinlin, 9 August 2023 in CLSGNU; 26♀43♂, China: Inner Mongolia prov., Tuquan County, Taihe,12 August 2023 in CLSGNU; 33♀50♂, China: Inner Mongolia prov., Horqin Right Middle Banner, Wutaiyingzi, 13 August 2023 in CLSGNU.

#### Type material of the synonyms examined.

*Coptotettixcircinihumerus*: ♀, holotype (Fig. 3A–C), China: Guangxi prov., Nandan County, Songhuahu, May 2003, in IZSNU.

*Coptotettixemeiensis*: ♀, holotype (Fig. 3D–F), China: Sichuan prov., Emeishan City, 13 June 2011, in IZSNU.

*Euparatettixrongshuiensis*: ♂, holotype (Fig. 4A–C), China: Guangxi prov., Rongshui County, 06 August 2003, in IZSNU.

*Euparatettixzayuensis*: ♀, holotype (Fig. 4D–F), China: Xizang, Zayu County, Menkong, 04–05 July 2005, in IZSNU.

*Macromotettixnigritubercle*: ♀, holotype (Fig. 5A–C) and 1♂ paratype, China: Guangxi prov., Fusui County, Bapan, 17 August 2004, in IZSNU.

*Macromotettixyaoshanensis*: ♂, holotype (Fig. 5D–F), China: Guangxi prov., Jinxiu County, Liula, 06 July 1997, in IZSNU.

*Tetrixalbistriatus*: ♀, holotype (Fig. 6A–C) and 1♂3♀ paratypes, China: Yunnan prov., Pingbian County, Daweishan, 26 July 2005, in IZSNU.

*Tetrixalbomaculatus*: ♂, holotype (Fig. 6D–F), China: Guangxi prov., Fusui County, Bapan, 17 August 2004, in IZSNU.

*Tetrixalbomarginis*: ♂, holotype (Fig. 7A–C), China: Yunnan prov., Dali County, Cangshan, 09 August 2004, in IZSNU.

*Tetrixcenwanglaoshana*: ♀, holotype (Fig. 7D–F), China: Guangxi prov., Tianlin County, Cenwanglaoshan, 31 May 2002, in IZSNU.

*Tetrixcliva*: ♂, holotype (Fig. 8A–C), China: Guangxi prov., Luocheng County, April 2003, in IZSNU.

*Tetrixduolunensis*: ♀, holotype (Fig. 8D–F) and 5♂7♀ paratypes, China: Inner Mongolia autonomous region, Duolun County, 29 June 1994, in IZSNU.

*Tetrixgrossovalva*: ♀, holotype (Fig. 9A–C), China: Jilin prov., Fongman County, Songhuahu, 19 July 1990, in IZSNU.

*Tetrixjiuwanshanensis*: ♀, holotype (Fig. 10) and 1♂ paratype, China: Guangxi prov., Rongshui County, 06 August 2003, in IZSNU.

*Tetrixlatipalpa*: ♂, holotype (Fig. 11A–C), China: Sichuan prov., Emeishan County, Mt. Emei, 16 August 2010, in IZSNU

*Tetrixliuwanshanensis*: ♂, holotype (Fig. 11D–F) and 1♂ paratype, China: Guangxi prov., lingshan County, Liuwanda Mountain, 24 August 2005, in IZSNU.

*Tetrixqinlingensis*: ♀, holotype (Fig. 12A–C) and 1♂1♀ paratypes, China: Shaanxi prov., Foping County, Zhongzui, 24 July 1997, in IZSNU.

*Tetrixrectimargina*: ♀, holotype (Fig. 12D–F), China: Guangxi prov., Tian’e County, Buliu River, 05 August 2002, in IZSNU.

*Tetrixxianensis*: ♀, holotype (Fig. 13C–E), China: Shaanxi prov., Xian City, 28 October 1990, in IZSNU.

*Tetrixxinchengensis*: ♂, holotype (Fig. 14A–C), China: Guangxi prov., Xincheng County, 14 July 2005, in IZSNU.

*Tetrixyunlongensis*: ♂, holotype (Fig. 14D–F), China: Yunnan prov., Yunlong County, 03 May 1998, in IZSNU.

#### Justification of the synonymies.

Holotype of *Coptotettixcircinihumerus* (Fig. 3A–C) from Guangxi and holotype of *Coptotettixemeiensis* (Fig. 3D–F) from Sichuan have a deformed frontal costa, and vertex and frontal costa forming an obtuse-rounded aspect in profile. These two taxa were misidentified as members of the genus *Coptotettix*: *Coptotettixcircinihumerus* has a widened vertex, a low pronotal median carina; antennal grooves inserted between inferior margins of compound eyes; hind wings extending beyond the apex of the pronotum; ventral margins of middle femora are slightly undulated. *Coptotettixemeiensis* is characterized by width of vertex between eyes 1.3 × wider than width of a compound eye; antennal grooves inserted between inferior margins of compound eyes; median carina of pronotum slightly arch-like before the shoulders and straight behind the shoulders in profile; hind wings nearly reach the apex of of the pronotum. *Coptotettixcircinihumerus* and *Coptotettixemeiensis* are completely consistent with the morphology of brachypronotal and brachypterous individuals of *T.japonica*.

*Euparatettixrongshuiensis* (Fig. 4A–C) from Guangxi was described on the basis of a single male holotype. The holotype has a deformed head, and the head is slightly exserted above the upper level of the pronotum. However, it is characterized by frontal ridge and vertex forming an obtuse angle; antennal grooves inserted between inferior margins of compound eyes; median carina of pronotum low and full length entire, in profile, slightly straight; ventral margins of middle femora straight; hind wings slightly surpassing apex of hind pronotal process. *Euparatettixzayuensis* (Fig. 4D–F) from Xizang was described based on two female specimens. The holotype has a deformed head, and the head is slightly exserted above the upper level of the pronotum. But it has a widened vertex, a low pronotal median carina, straight and widened middle femora. These two taxa were misidentified as members of the genus *Euparatettix*. *E.rongshuiensis*, and *E.zayuensis* appear to be conspecific with *T.japonica* (brachypronotal and brachypterous individuals).

*Macromotettixnigritubercle* (Fig. 5A–C) from Guangxi represents a synonym of *T.japonica*. It is the same as *T.japonica* (brachypronotal and brachypterous forms) in all of the characters except for the slightly obliquely truncate posterior angles of the lateral lobes of the pronotum. The slightly truncate posterior angles of lateral lobes of pronotum fit the known variability of *T.japonica*. Examination of *Macromotettixyaoshanensis* (Fig. 5D–F) from Guangxi, shows that this specimen is a brachypronotal and brachypterous *T.japonica*. It is characterized by head and eyes not exserted above pronotal surface; frontal ridge and vertex forming an obtuse angle; antennal grooves inserted between inferior margins of compound eyes; median carina of pronotum low and full length entire; hind wings slightly surpassing apex of hind pronotal process. The specimen also has interhumeral carinae between shoulders, while the interhumeral carinae are inconspicuous and small.

*Tetrixalbistriatus* (Fig. 6A–C) from Yunnan, which completely fits the morphology of brachypronotal and brachypterous individuals of *T.japonica*, also has a white stripe on the median carina of the pronotum. The two taxa are conspecific and characterized by a frontal ridge and vertex forming an obtuse angle; median carina of pronotum nearly straight in profile, slightly arcuate in forepart; lower margins of mid femora slightly straight, width of mid femora equal to width of tegmina in females; hind wings not reaching the apex of the hind pronotal process.

*Tetrixalbomaculatus* (Fig. 6D–F) from Guangxi was described based on a single male holotype. This specimen representing a brachypronotal and brachypterous *T.japonica*, has interhumeral carina between the shoulders and white spots behind the shoulders. It is characterized by head and eyes not exserted above pronotal surface; frontal ridge and vertex forming an obtuse angle; antennal grooves inserted between inferior margins of compound eyes; lower margins of mid femora straight, width of mid femora equal to width of tegmina in females. *Tetrixalbomarginis* (Fig. 7A–C) from Yunnan was also described based on a single male holotype. This specimen is similar to the morphology of brachypronotal and brachypterous individuals of *T.japonica*, and has white pronotal margins and a slightly elevated median carina of the pronotum. But it has a widened vertex, a low pronotal median carina, straight ventral margins of middle femora, as well as shortened pronotum and hind wings.

*Tetrixcenwanglaoshana* (Fig. 7D–F) from Guangxi was described based on a single female holotype. It is conspecific with *T.japonica* (pauropronotal individuals) and has a widened vertex, frontal ridge and vertex forming an obtuse angle in profile, low and full length entire pronotal median carina, as well as extended hind wings and pronotum.

*Tetrixcliva* (Fig. 8A–C) from Guangxi was described based on a single male holotype. In the original description, the specific epithet *cliva* refers to the shape of the upper margin of the pronotum, in profile, with a triangular process before the shoulders. Examination of holotype showed that the shoulders of pronotum were compressed and were deformed before the shoulders to create a triangular protuberance. Other important traits are the same as *T.japonica*: it has straight ventral margins of middle femora, as well as shortened pronotum and hind wings; frontal ridge and vertex forming an obtuse angle; antennal grooves inserted between inferior margins of compound eyes. Therefore, *T.cliva* is conspecific with *T.japonica* (brachypronotal and brachypterous individuals).

We examined the type series of *Tetrixduolunensis* (Fig. 8D–F) from Inner Mongolia. The anterior margin of the fastigium of the vertex was slightly arcuate and slightly surpassed the anterior margin of the eye in some individuals. However, most individuals completely fit the morphology of brachypronotal and brachypterous individuals of *T.japonica*. It has a widened vertex, straight frontal costa, low pronotal median carina, and extended hind wings and pronotum. Thus, *T.duolunensis* is considered to be a synonym of *T.japonica*.

*Tetrixgrossovalva* (Fig. 9A–C) from Jilin was described based on a single female holotype that appears to be conspecific with *T.japonica* (pauropronotal individuals). It has a widened vertex, straight frontal costa, low and full length entire pronotal median carina, and extended hind wings and pronotum.

*Tetrixjiuwanshanensis* (Fig. 10) from Guangxi completely fits the morphology of pauropronotal individuals of *T.japonica*. These two taxa are conspecific and characterized by the frontal ridge and vertex forming an obtuse angle; width of vertex wider than width of an eye, 1.6 ×; median carina of pronotum nearly straight in profile, slightly arcuate in forepart; ventral margins of mid femora slightly undulated, width of mid femora equal to width of tegmina in females.

*Tetrixlatipalpa* (Fig. 11A–C) from Sichuan was described based on a single male holotype. The holotype has a deformed pronotum, and the median carina is slightly elevated and slightly sinuate. It is characterized by the frontal ridge and vertex forming an obtuse angle; width of vertex wider than width of an eye 1.6 ×; ventral margins of mid femora straight, width of mid femora equal to width of tegmina in females; the hind wings distinctly surpass the apex of the hind pronotal process. It is conspecific with brachypronotal and brachypterous *T.japonica*.

*Tetrixliuwanshanensis* (Fig. 11D–F) from Guangxi completely fits the morphology of brachypronotal and brachypterous individuals of *T.japonica* except for the slightly obtuse anterior margin of the pronotum. The slightly obtuse anterior margin of the pronotum fits the known variability of *T.japonica*. It is characterized by width of vertex between eyes is 1.1 × the width of the compound eye; frontal ridge and vertex forming an obtuse angle; antennal grooves inserted between inferior margins of compound eyes; median carina of pronotum slightly arcuate in forepart; ventral margins of mid femora straight, width of mid femora wider than width of tegmina.

*Tetrixqinlingensis* (Fig. 12A–C) from Shaanxi is conspecific with *T.japonica* (pauropronotal individuals). These two taxa are characterized by width of vertex between eyes wider than width of compound eye; frontal ridge and vertex forming an obtuse angle; median carina of pronotum nearly straight in profile, slightly arcuate in forepart; ventral margins of mid femora slightly undulated, width of mid femora slightly narrower than width of tegmina in female; hind pronotal process surpassing apex of hind femur; hind wings surpassing apex of hind pronotal process.

*Tetrixrectimargina* (Fig. 12D–F) from Guangxi was described based on a single female holotype. *Tetrixrectimargina* represents a synonym of *T.japonica*. It is the same as brachypronotal and brachypterous *T.japonica* in all the characters except for the narrow vertex (width of vertex between eyes is equal to the width of compound eye). The slightly narrow vertex fits the known variability of *T.japonica*.

*Tetrixruyuanensis* (Fig. 13A–C) from Guangdong was described based on a single female holotype, according to the description of the species by Liang and Zheng (1998), *T.ruyuanensis* is very similar to brachypronotal and brachypterous individuals of *T.japonica*. The only difference is median carina of pronotum arcuate before shoulders in profile in *T.ruyuanensis* (vs median carina of pronotum nearly straight before shoulders in profile in *T.japonica*). Topotypes of *T.ruyuanensis* were examined, and it was found that some individuals have an arcuate median carina before shoulders (Fig. 13B). However, most individuals have a nearly straight median carina (Fig. 13A). Thus, *T.ruyuanensis* is considered to be a synonym of *T.japonica*.

*Tetrixxianensis* (Fig. 13D–F) from Shaanxi was described based on a single female holotype, and *T.xianensis* represents a synonym of *T.japonica*. It is the same as brachypronotal and brachypterous *T.japonica* in all the characters except for the slightly arcuate vertex and slightly extended hind wings and pronotum. The slightly arcuate vertex and slightly extended hind wings and pronotum fit the known variability of the species.

*Tetrixxinchengensis* (Fig. 14A–C) from Guangxi was described based on a single male holotype, and *T.xinchengensis* represents a synonym of *T.japonica*. It is the same as brachypronotal and brachypterous *T.japonica* in all of the characters except for the slightly extended hind wings and pronotum. Extended hind wings and pronotum are consistent with the known variability of *T.japonica*.

*Tetrixyunlongensis* (Fig. 14D–F) from Yunnan was described based on a single male holotype, and *T.yunlongensis* represents a synonym of *T.japonica*. It is the same as brachypronotal and brachypterous *T.japonica* in all morphological characters except for the slightly obtuse anterior margin of the pronotum. The slightly obtuse anterior margin of the pronotum is consistent with the known variability of *T.japonica*. It is characterized by width of vertex between eyes is 1.4 × the width of the compound eye; frontal ridge and vertex forming an obtuse angle; antennal grooves inserted between inferior margins of compound eyes; median carina of pronotum slightly arcuate in forepart; ventral margins of mid femora straight.

Type material of *Tetrixzhoushanensis* from Zhejiang was not examined, but according to the original description and photographs (Fig. 15) of the type specimens in Gao et al. (2022), *Tetrixzhoushanensis* is identical to the morphology of brachypronotal and brachypterous individuals of *T.japonica*. *Tetrixzhoushanensis* has a narrower vertex (width of vertex between eyes is 1.1 × the width of the compound eye) than typical *T.japonica*, but this trait should be studied more in future in order to see if it has maybe a subspecies value. It is characterized by antennal grooves inserted between inferior margins of compound eyes; median carina of pronotum slightly arcuate in forepart; ventral margins of mid femora straight.

**Figure 16.**
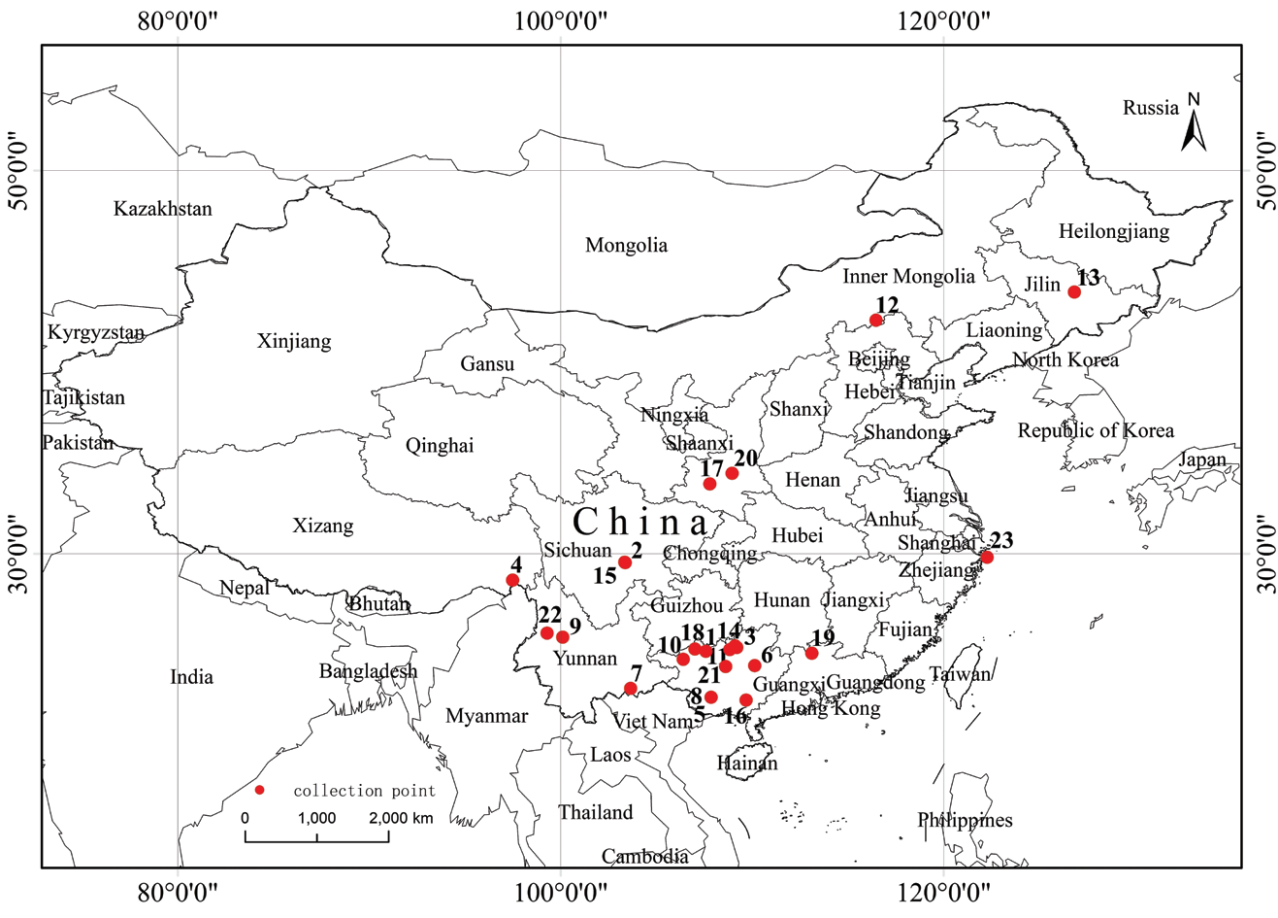
Distribution map of *Tetrixjaponica* with all type localities of all the synonyms. 1 *C.circinihumerus*; 2 *C.emeiensis*; 3 *E.rongshuiensis*; 4 *E.zayuensis*; 5 *M.nigritubercle*; 6 M.yaoshanensis; 7 *T.albistriatus*; 8 *T.albomaculatus*; 9 *T.albomarginis*; 10 *T.cenwanglaoshana*; 11 *T.cliva*; 12 *T.duolunensis*; 13 *T.grossovalva*; 14 *T.jiuwanshanensis*; 15 *T.latipalpa*; 16 *T.liuwanshanensis*; 17 *T.qinlingensis*; 18 *T.rectimargina*; 19 *T.ruyuanensis*; 20 *T.xianensis*; 21 *T.xinchengensis*; 22 *T.yunlongensis*; 23 *T.zhoushanensis*.

## ﻿Discussion

*Tetrixjaponica* is widely distributed in East Asia, and China, where is very common except in Hainan and Xinjiang. The People’s Republic of China has a vast and complex ecological environment with an abundance of insect species. It has a huge diversity of Tetrigidae and has the most described species in the world (e.g., Liang and Zheng 1998, Zheng 2005a, Cigliano et al. 2023). However, 23 species are herewith synonymized with *T.japonica* because their descriptions were not based upon valid differences, but a wrongly assessed species variability, often caused by too few specimens being examined. Among the 23 synonyms, 11 synonyms (*E.rongshuiensis*, *T.cliva*, *T.latipalpa*, *T.albomaculatus*, *T.cenwanglaoshana*, *T.grossovalva*, *T.xinchengensis*, *T.rectimargina*, *Tetrixruyuanensis*, *T.xianensis*, *T.yunlongensis*) were based on single specimens. One should be very careful when describing new Tetriginae species because few individuals can lead to wrong conclusions or a taxonomic inflation.

Additionally, some deformities were observed in the single-specimen species. The main causes of specimen deformities were recorded were two-fold. One was that the body was compressed by external forces during growth (*T.cliva*, *T.latipalpa*, *C.circinihumerus*, *C.emeiensis*). The other is that deformities were caused by humans during specimen preparation. For example, pygmy grasshopper specimens are usually dried and pinned. When inserting a pin into a specimen, the pin typically passes through the right side of the thorax from the shoulder of pronotum, which tends to push the pronotum down. This elevates the previously non-protruding heads such as in *E.rongshuiensis* and *E.zayuensis*.

Some pygmy grasshoppers are highly polymorphic in both colors and markings. *Tetrixjaponica*, which occurs in both grass and sand microhabitats, exhibits large variations in body coloration and pronotum markings. Within a single population, the basal body coloration can vary from blackish brown to yellowish brown to pale grey. Some *T.japonica* are bi-colored, with whitish and blackish markings on the dorsal surface of the pronotum. In contrast, some *T.japonica* have no markings, whereas others have spots on the pronotum. The number, shape, and position of spots also varies among the spotted morphs (Tsurui et al. 2010). Therefore, in the classification of Tetrigoidea, and especially in Tetriginae: Tetrigini the spots on the dorsal pronotum are not a reliable taxonomic feature. In Batrachideinae and Metrodorinae, some group coloration is helpful (Tumbrinck and Skejo 2017; Kasalo et al. 2021; Itrac-Bruneau and Doucet 2023), but these groups are exceptions within Tetrigidae, not the rule. In the early classification of Tetrigoidea in China (Liang and Zheng 1998; Zheng 2005a), this character was often used as a taxonomic feature. As a result, *T.albistriatus*, *T.albomaculatus*, and *T.albomarginis* have been synonymized with *T.japonica*.

Dimorphism in wing length is known in many insect species, and in some species of pygmy grasshoppers, both the hind wings and the pronotum can be dimorphic (Deng 2021; Zhang et al. 2022). Because pygmy grasshoppers can vary in pronotum characters and length of the hind wings (Deng 2021; Zhang et al. 2022), the length of the hind wings and the pronotum cannot be used as taxonomic characters for classification of some species. However, in previous classifications of Tetrigoidea in China (e.g., Liang and Zheng 1998; Zheng 2005a; Deng et al. 2007a), these two characters were often used alone as diagnostic features; the dimorphism of these two characters was not considered. Therefore, misidentification of some species of *Tetrix* can readily occur and lead to synonyms, such as *T.cenwanglaoshana*, *T.grossovalva*, *T.jiuwanshanensis*, *T.qinlingensis*, and *T.xinchengensis*, which have all been here synonymized with *T.japonica*.

Similarly, *T.japonica* individuals can also exhibit variability in the shape of posterior angles of lateral lobes of pronotum and fastigium of vertex and anterior margin of pronotum, height median carina of pronotum, width of vertex between eyes, width of the middle femur, length of body, length of pronotum, and length of hind femur (Table 1). Therefore, these features alone cannot be used separately in taxonomic identification. Hence, when *T.japonica* is identified among other *Tetrix* species, we are strongly recommended using a combination of characters: head and eyes not exserted above pronotal surface; width of vertex between eyes generally wider than or sometimes equal to width of a compound eye; anterior margin of fastigium of vertex truncated or slightly arcuate and slightly surpassing anterior margin of eye; frontal ridge and vertex forming an obtuse angle; antennal grooves inserted between inferior margins of compound eyes; anterior margin of pronotum generally truncate; median carina of pronotum low and full length entire, in profile, slightly straight or slightly arch-like before the shoulders and straight behind the shoulders; ventral margins of middle femora straight or slightly undulated; with tegmina and hind wings developed, nearly reach apex of hind process or more.

The problematic taxonomy of *T.japonica* suggests that similar problems will occur in other species of *Tetrix*. This genus requires more research, especially, regarding interspecific and intraspecific variability.

## Supplementary Material

XML Treatment for Tetrix japonica

## ﻿Additional information

### Author ORCIDs

Ying Long https://orcid.org/0009-0003-8226-8958

Caili Teng https://orcid.org/0009-0008-2124-959X

Chaomei Huang https://orcid.org/0000-0003-1046-1348

Rongjiao Zhang https://orcid.org/0000-0001-5545-856X

Weian Deng https://orcid.org/0000-0002-8023-2498

Liliang Lin https://orcid.org/0000-0002-9972-4732

### Data availability

All of the data that support the findings of this study are available in the main text.
